# Evaluation of three therapeutic alternatives for the early treatment of ovine pregnancy toxaemia

**DOI:** 10.1186/s13620-015-0053-2

**Published:** 2015-10-24

**Authors:** L. Cal-Pereyra, J. R. González-Montaña, A. Benech, J. Acosta-Dibarrat, MJ. Martín, S. Perini, MC. Abreu, S. Da Silva, P. Rodríguez

**Affiliations:** Pathology Department, Veterinary Faculty, University of La República, Montevideo, Uruguay; Medicine, Surgery and Anatomy Veterinary Department, Veterinary Faculty, University of León, León, 24071 Spain; Veterinary Hospital, Veterinary Faculty, University of La República, Montevideo, Uruguay; Center for Research and Advanced Studies in Animal Health, Faculty of Veterinary Medicine, Autonomous University of Mexico State, Toluca, Mexico

**Keywords:** Pregnancy toxaemia, ß-hydroxybutyrate, Glycaemia, Glycerol, Propylene glycol, Sheep

## Abstract

**Background:**

Ovine pregnancy toxaemia is a common metabolic disorder of ewes due to increased foetal energy requirements in late pregnancy. This pathology is a metabolic condition characterized by hypoglycaemia and hyperketonaemia resulting in the inability of the animal to maintain an adequate energy balance. The response to treatment is effective, if it is started in the early stages of the disease, when irreversible neurological injuries have not yet been established. The aim was to evaluate three therapeutic alternatives to effectively reverse the disease process in its early stages.

For this, thirty adult Corriedale ewes, pregnant with a single lamb, were randomly separated in three groups of ten animals each, at day 130 of gestation. From that day onwards, ewes were locked up for forage fasting until glycaemia reached clinical values defining sub-clinical pregnancy toxaemia (1.59 ± 0.24 mmol/L). After fasting, ewes grazed and received a treatment for 4 days: 50 ml *i.v.* infusions of *hypertonic glucose* and 20 UI insulin/ewe/day s.c. or 100 ml/sheep/12 h of glycerol together with propylene glycol oral solution or fed with pasture supplemented with two daily intakes 300 g/sheep of cracked corn. Glycaemia and ß-hydroxybutyrate were determined in all the animals from the beginning of fasting until the completion of the treatment.

**Results:**

Fasting caused a decline in blood glucose in the 3 groups. This decline continued until fasting was withdrawn and treatment began. Thereafter blood glucose increased in all three groups, although in the group supplemented with glycerol and propylene glycol it started to increase significantly after 12 h. The values of ß-hydroxybutyrate decreased in the 3 groups at the start of treatment, and this decline was more pronounced earlier on and in the group supplemented with glycerol and propylene glycol. We found no significant differences between all experimental groups. No animal showed clinical signs of pregnancy toxaemia throughout the research.

**Conclusions:**

The three treatments administered to sheep affected by sub-clinical pregnancy toxaemia were able to restore normal concentration of glucose and ß-hydroxybutyrate in blood, although *per os* administration of 100 ml/sheep/12 h of glycerol with propylene glycol, was the most successful treatment, normalizing the aforementioned biochemical parameters in a shorter time.

## Background

Ovine pregnancy toxaemia (OPT) is a common metabolic disorder of undernourished ewes due to increased foetal energy requirements in late pregnancy [1]. This pathology is characterised by hypoglycaemia and hyperketonaemia resulting in the inability of the animal to maintain an adequate energy balance [2–5]. The disease has a significant economic impact on sheep and goat enterprises due to loss of sheep and foetuses; veterinary costs and dam balance [3–7]. The disease has been associated to multiple gestation [2, 7–9], but it can be found in ewes carrying a singleton, when winters are rigorous and nutritional deficiencies occur [10, 11] or during periods of starvation after excessive fatness [8, 12] .

It has been shown that fasting causes a rapid triacylglycerides (TAG) mobilization from adipose tissue, which is reflected in the rapid rise of non-esterified fatty acids (NEFA) values representing earlier blood changes in sheep under fasting. This increase in lipid mobilization leads to a high incidence of hepatic steatosis in these animals [10, 13].

One of the most important and measurable factors to determine whether a sheep suffers the disease or not, is the evaluation of the hepatic function. The liver is important for the blood glucose metabolism, for the glucose’s tissue supply and because it is virtually the only organ where the gluconeogenesis takes place; although there are small contributions from the kidney [5, 9, 12].

Early detection of OPT in susceptible animals is therefore essential for a successful treatment [10, 14]. Treatments described for pregnancy toxaemia outcomes vary and turn out to be costly when the disease affects a high number of animals. The response to treatment is effective, if started in the early stages of the disease, when irreversible neurological injuries have not been established, and when the animal is not yet in decubitus [10, 12, 15]. An early disease diagnosis is important, because it will allow a rational and effective treatment [10]. The priority objective in the treatment of the disease is to increase the formation of glucose and its utilization at tissue level, and it must also increase the use of the ketone bodies; which to solve or minimise the acidosis and hydroelectrolytics disorders [12].

The aim of this work was to test three therapeutic alternatives to effectively reverse the disease process in early stages for application in extensive sheep production systems where the cost/benefit ratio is extremely important.

## Methods

Fifty-five Corriedale adult ewes aged 4–6 years were randomly selected from a flock under usual extensive production conditions. At the beginning of the experiment, all ewes were in similar physicial conditions; scores were between 2.5 and 3.5 on a 1–5 scale [16] and the mean bodyweight was 54.2 ± 5.7 kg. The oestrous cycles of ewes were synchronized using intravaginal devices with 60 mg of medroxiprogesterone (Sincrovin, Santa Elena, Uruguay) inserted for 12 days, and then the ewes were left with two fertile rams for 4 days until pregnancy was achieved. The mating date was recorded as day 0 of gestation and was confirmed with transabdominal ultrasonography between days 40 and 50 of gestation. Corriedale sheep mean gestational length is reported to be 149 days [17]. Afterwards, 30 ewes carrying a single foetus were included in the protocol. Sheep with a single foetus were chosen to avoid or minimise the occurrence of spontaneous pregnancy toxaemia.

Ewes were grazed on pasture composed mainly by *Cynodon dactylon.* Every 100 g of grass dry matter provided 8.72 % crude protein and 1.86 Mcal EM/kg dry matter metabolisable energy (Laboratory of Nutrition Faculty of Veterinary Medicine, University of la República, Uruguay).

On day 130 of gestation, sheep were randomly divided into three groups: A, B and C of 10 animals each. From this moment, sheep were penned in roofed paddocks with a concrete floor and ad libitum access to water. They were under total forage fasting until glycaemia reached clinical values of sub-clinical pregnancy toxemia (1.59 ± 0.24 mmol/L) [18]. After reaching these blood glucose values, fasting was withdrawn individually; and sheep were reintroduced into the natural pasture. All sheep, and for 4 days, received the corresponding treatment assigned to each group:

Firstly, group A ($\overline{x}$ = 55.75 ± 9.0 Kg) animals received first 50 ml of 50 % i.v. infusions of hypertonic glucose (Dextrolena®, Santa Elena, Uruguay) and lastly, in addition, 20 UI subcutaneous insulin infusion, daily per ewe (Caninsulin®, Intervet, Argentina). This treatment was administered after the collection of blood samples at 06:00 am.

Group B ($\overline{x}$ = 54.38 ± 8.5 Kg). Was administered to each sheep every 12 h 100 ml of the oral solution Acetolena® (Santa Elena, Uruguay), composed of 700 g of glycerol and 200 g of propylene glycol each liter. This treatment was administered after blood samples collection at 06:00 am and 06:00 pm.

Group C ($\overline{x}$ = 53.4 ± 8.1 Kg). After the collection of blood samples, at 06:00 am and 06:00 pm each sheep was fed two daily intakes of pasture supplemented with 300 g. of cracked corn.

Blood was collected by jugular venipuncture using an 18G needle attached to a 10 ml syringe. From confinement (started on day 130 of pregnancy) and until treatment completion all sheep were bled daily at 06:00 am to asses ß-hydroxybutyrate (BOHB) and every 12 h (06:00 am and 06:00 pm) to determine glycaemia. Blood for glycaemia determination was collected in tubes containing sodium fluoride, while for BOHB determination blood was collected in dry tubes. Glycaemia determination was performed within two hours of the serum being collected, and to determine BOHB frozen samples we stored at - 20 ° C in Eppendorf tubes properly labelled and identified until processing.

We used Ranbut® commercial kits (Antrim, UK) and Glucose Liquicolor® (Wiesbaden, Germany) for BOHB and glycaemia determinations, with the aid of a digital Humalyser Junior colorimeter at 37 °C, measuring 500 and 330 nm respectively.

The experimental procedures were conducted under field conditions at the Faculty of Veterinary Medicine, University of la Republica, Uruguay, located in Libertad, Department of San José, Uruguay (34° 38’S; 56° 39´W), upon approval of the local Animal Welfare Committee (Honorary Committee for Animal Experimentation).

### Statistical analysis

Statistical analysis was carried out with Statistica 6.0 (Stat Soft Inc, Tulsa, OK, USA). Data distribution was considered normal when p > α using the Shapiro-Wilk test. ANOVA was used to evaluate statistical differences between treatment groups for blood metabolites. When differences were observed, a Tukey test was performed. Differences were considered significant when *p* < 0.05.

## Results

**Fig. 1:**
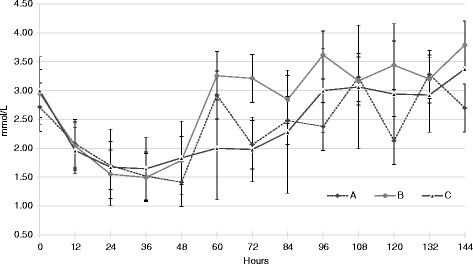
Glycaemia evolution in sheep. Glycaemia ($\overline{x}$ ± ds) mmol/L obtained in sheep from groups A (glucose and insulin), B (G + PG) and C (corn) every 12 h from day 130 of pregnancy throughout treatment. Hours: 0 fast starts; 36 fast ends; 48 treatments start

Three experimental groups were formed on day 130 of pregnancy, glycaemia in the sheep did not show statistic difference among the three groups. Average glycaemia for groups A; B and C, at this stage, were: 2.71 ± 0.66; 2.95 ± 0.57 and 3.00 ± 0.57 mmol/L, respectively. Figure 1 shows that glycaemia in the three groups started decreasing at 0 h (fasting beginning). This decreasing continued for 36 h, when the sheep stopped fasting, since all groups showed glycaemia levels compatible with sub-clinical toxaemia, showing no significant differences among groups at this time (1.52 ± 0.49; 1.49 ± 0.54 y 1.64 ± 0.55 mmol/L, for groups A, B y C, respectively).

Once the treatments started (48 h after treatment began) glycaemia values rose in the three groups. In the sheep of group B, glycaemia increased earlier and values were higher than in the other groups throughout the treatment (Fig. 1). Glycaemia in this group showed a significant difference (*p* < 0.001) from glycaemia in group C, at 60 h of confinement (12 h after treatment had started), and it remained like this for 12 more hours, until 72 h (Table 1). In comparison, glycaemia in group A increased earlier than in group B, although this difference became significant in samples taken at 72 h (24 h after the treatment was implemented) (*p* < 0.0001). After 24 h treatment was implemented in group A, these two groups showed significant difference in glycaemia values, from this stage until treatment completion, although they did not show statistical difference in samples taken 12 h after treatment implementation in group A (Table 1). When comparing glycaemia in groups A and C, they only showed a significant difference after 60 h (12 h after therapy started) (*p* < 0.01), without showing differences until the last sampling (*p* < 0.001) (Table 1).

**Table 1:** Glycaemia evolution in three groups

Hours	Group A	Group B	Group C
0	2.71 ± 0.66	2.95 ± 0.57	3.00 ± 0.59
12	2.08 ± 0.64	2.04 ± 0.84	1.96 ± 0.40
24	1.70 ± 0.74	1.55 ± 0.54	1.67 ± 0.66
36	1.52 ± 0.49	1.49 ± 0.54	1.64 ± 0.55
48	1.41 ± 0.48	1.79 ± 0.75	1.83 ± 0.64
60	2.92 ± 0.64 c	3.26 ± 0.48 a	2.00 ± 0.89 b
72	2.06 ± 0.67 a	3.21 ± 0.58 b	1.98 ± 0.56 a
84	2.48 ± 1.01	2.84 ± 0.48	2.28 ± 1.07
96	2.38 ± 0.54 b	3.62 ± 0.68 c	3.00 ± 0.72
108	3.23 ± 0.89	3.17 ± 0.29	3.06 ± 1.07
120	2.13 ± 0.82 b	3.44 ± 0.61 c	2.94 ± 1.22
132	3.28 ± 1.12	3.20 ± 0.49	2.92 ± 0.64
144	2.70 ± 0.42 a	3.79 ± 0.42 b	3.37 ± 0.03 b

Glycaemia ($\overline{x}$ ± ds) mmol/L obtained in sheep from group A (glucose and insulin); B (G + PG) and C (corn) every 12 h from day 130 of pregnancy throughout the treatment. Statistical differences: ^a-b^: *p* < 0.001; ^b-c^: *p* < 0.01

**Fig. 2:**
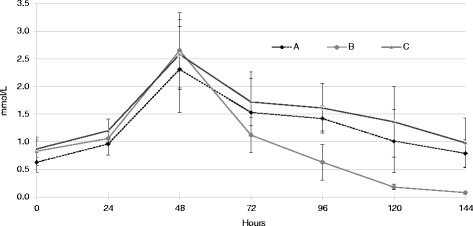
ß-hydroxybutyrate evolution in sheep. ß-hydroxybutyrate (BOHB) ($\overline{x}$ ± ds) mmol/l obtained from sheep in groups A (glucose and insulin), B (G + PG), and C (Corn) every 24 h from day 130 of pregnancy until the end of treatments. Hours: 0 fast starting time; 48 treatments starting time

BOHB values in sheep of the three groups, did not show significant differences on day 130 of pregnancy, average values in groups A; B and C were: 0.63 ± 0.39; 0.83 ± 0.53 and 0.87 ± 0.32 mmol/L, respectively. From this stage on, BHOB started increasing in the three groups until treatment began. When removing animals from fasting, values of these ketone bodies did not show significant differences among the groups (2.32 ± 0.53; 2.54 ± 0.53 and 2.62 ± 0.,80 mmol/L respectively). BOHB started decreasing in the three groups, once treatment began, although the decrease was sharper and earlier in group B. The differences found between groups A and B (*p* < 0.01) and C and B (*p* < 0.05) started at 48 h from treatment initiation, remaining like this until treatment finished. BOHB serum values between groups A and C however did not show significant differences throughout treatment (Fig. 2).

No animal showed clinical signs of pregnancy toxaemia throughout the research, nor during the period of fasting, nor the phase of treatment.

## Discussion

Considering the BOHB and glycaemia values, at the moment of removing the sheep from fasting and the fact that there was no evidence of any clinical signs, it is reasonable to assume that these animals were affected by sub-clinical pregnancy toxaemia before treatments started [18, 19].

Early diagnosis is very important. The simplest method is the use of test strips. While it is important to bear in mind that the test strips used for the pregnancy toxaemia diagnosis is based on the semi-quantitatively determination of ketone bodies in urine, nevertheless these stripes react in presence of acetoacetate and acetone and do not detect BOHB which is found in larger quantity in this disease. The positive reaction of these test stripes clearly indicates the presence of ketonuria. However, there have recently appeared on the market portable and electronic devices (FreeStyle®, Precision Xceed®, Precision Xtra®, etc.) with great field level diagnostic potential, specially for veterinary clinicians, measuring blood BOHB in sheep [20], goats [21] or cattle [22]. There has been found a high correlation between the BOHB and glucose values measured with these devices and the ones obtained by the laboratory, showing high sensitivity and specificity [20, 22].

Taking into account that one of the main objectives of the treatment for pregnancy toxaemia is the increase of glycaemia [12, 14, 15, 23], when assessing the results in this paper we observed that once treatment started glycaemia began increasing in the three experimental groups. However, in sheep treated with propylene glycol and the ones treated with an i.v. infusion of glucose plus insulin, this increase took place earlier than in sheep treated with corn supplementation. This difference can be explained if we consider the different rates of absorption and conversion of the precursors used into glucose for treatment in groups A and B. According to Herdt and Emery [23] an i.v. infusion of glucose immediately causes an increase in blood glucose concentration resulting in transient hyperglycaemia, which we did not observe in our research as a consequence of the insulin subcutaneous infusion immediately after the glucose infusion, since this promotes the rapid glucose uptake and utilization by peripheral tissues [14].

Glycaemia increase in response to propylene glycol after 12 h of treatment from 1.79 ± 0.75 to 3.26 ± 0.48 mmol/L. Propylene glycol, which is mainly absorbed intact directly from the rumen at a rate of 40 % per hour [23] and reaches its maximum blood level within 30 min of administration and maximum blood glucose conversion at about 4 h after administration. Propylene glycol transformation in glucose probably occurs via conversion to pyruvate [23].

In this group, blood glucose values were the highest throughout the treatment. Propylene glycol produces a rapid increase of glucose, while glycerol is slowly degraded in the rumen producing a high proportion of propionate, main precursor of glucose via gluconeogenesis, resulting in a glycaemia increase for a relatively long period and furthermore, glycerol and propylene glycol treatment was repeated twice daily according to Rook [5], Wierda et al. [24] and Sienra et al. [25], all of which can explain glucose behaviour

In the group where fed corn (group C), glycaemia increased from 1.83 ± 0.64 to 2.00 ± 0.89 mmol/L, after 12 h of the start of the treatment, reaching 3.00 ± 0.72 mmol/L 48 h later. Ruminal microorganisms must attack the corn and transform it into volatile fatty acids, especially propionic acid [26, 27], which once formed, has to be absorbed at ruminal papillae level, being partly transformed in lactate in the rumen wall and both are converted into glucose in the liver via neoglucogenesis. Between 18 and 42 % starch from corn may escape rumen degradation and be digested in the small intestine [28]. However, only 30 to 35 % of the glucose formed from starch intestinal digestion, can be found in the portal vein [29].

The treatment of the sheep with intravenous glucose did not reach stable glycaemia concentrations, rapid increases post-administration were produced, followed by important decreases thereof. González-Montaña et al. [30] and González-Montaña et al. [31] described this glycaemia behaviour and attributed it to glucose renal excretion when it was administered intravenously. Intravenous infusions of glucose solutions is bound to result in transient hyperglycemia leading to diuresis and urinary loss of a large portion of the administered glucose [23]. Fox [32] established that after an i.v. infusion of glucose, approximately an 80 % of the dose is excreted by urine.

Considering that another of the objectives of pregnancy toxaemia treatment is to restore blood ketone bodies to normal concentrations [12, 23], results in this research showed that while blood BOHB decreased in the three experimental groups, once treatments were implemented, that decrease was sharper and earlier than in sheep treated with propylene glycol, and in addition BOHB remained significantly lower throughout the research. We think that the fatty acids metabolism was altered suppressing its mobilization from the adipose tissue, reducing their entry into the liver and reducing their transformation into mitochondrias. Propylene glycol increases pyruvate concentration with the subsequent oxaloacetate production via pyruvate carboxylase. Available oxaloacetate increase is expected to produce an increase in intramitochondrial citrate concentration. Intramitochondrial citrate escapes to form malonyl-CoA when is increased, it is a powerful transformation suppressor of fatty acids into mitochondria [33–35]. The fatty acids entry reduction into hepatic mitochondrias results in hepatic ketosis decreasing. Additionally, glucose supply provided by propylene glycol treatment may increase the insulin:glucagon relationship therefore affecting ketosis [23].

Glucose infusion results in a gluconeogenesis reduction [23], leading to an increase of the Krebs cycle which intermediates concentration inside the mitochondria due to the smaller quantity of substrates that are transported to the cytosol serving as glucose precursors. Citrate is one of those intermediaries, when increases it also increases Malonyl-CoA formation [33–35]. Herdt and Emery [23] also add that a glucose injection may cause a reduction in glucagon plasma since its secretion decreases in response to hyperglycaemia.

Increased citrate (and malonyl-CoA) and glucagon reduction will cause the fatty acid entry reduction inside the hepatic mitochondria. To these effects in the ketone bodies synthesis reduction we must add the antiketogenic effects of insulin, which was administered together with the intravenous glucose. The antiketogenic effect of insulin causes the following actions to occur: 1) it depresses the release of fatty acids from adipose tissue; 2) it promotes the use of ketone bodies in peripheral tissues; 3) it suppresses the entry of fatty acids to the liver; 4) relatively low insulin concentrations block the ketogenic effect of glucagon, thus limiting the fatty acids entry into the mitochondria [23, 36].

While the three treatments applied were able to restore blood glucose and BOHB normal concentrations, glycerol + propylene glycol treatment was the one, which achieved the results in less time and it had a more prolonged effect. Propylene glycol treatment results administered in the same doses and frequencies as in this research, in animals with clinical pregnancy toxaemia showed contradictory results. While Sienra et al. [25] indicated good results and metabolic parameters normalization, Wierda et al. [24] only achieved good results in mild forms of the disease, reporting that in animals with advanced pregnancy toxaemia results were poor. Sargison et al. [1] and Brozos et al. [14] suggested that although given a complete treatment, only one third of sheep with clinical pregnancy toxaemia would probably survive Koenig and Contreras [37] reported that in sheep induced to toxaemia by fasting, propylene glycol treatment reduced mortality in about 50 %. The differences found are explained by considering Rook’s proposals [5]. This author suggested that precursors compounds of glucose synthesis in the liver would be useful provided there is no organ severely compromised. Herdt and Emery [23] added that the ability to use propylene glycol is reduced in liver fatty infiltration. According to our findings and to Marteniuk and Herdt [15], the treatment with propylene glycol should be initiated as soon as possible, before as complications of the disease appear (acidosis, irreversible neurological damage, severe dehydration, kidney failure, etc.) and the chances of recovery would be lower.

Regarding corn treatment we can make the same suggestion considering the results in this research. Marteniuk and Herdt [15] stated that sheep in early stages of the disease and still maintaining their appetite, the energy increase supplied by the starch in the diet would be enough to reverse the conditions, adding that a more vigorous treatment is needed when animals are not eating or are eating very little. This type of treatment would be of practical use in subclinical pregnancy toxaemia cases in a commercial flock.

Koenig and Contreras [37], Cal Pereyra et al. [10] and Lima et al. [7] noted that advanced cases of clinical pregnancy toxaemia caused hyperglycaemia, thus glucose based therapy would have no effect at that stage of pregnancy. In spite of this fact, our findings show that at the early stages of pregnancy, treatment with intravenous glucose and subcutaneous insulin in pregnancy toxaemia is useful to reverse the process. However, according to Rook, [5] in considering the usage of this treatment, it would be of little practical use in cases of pregnancy toxaemia in conventional flocks but it would be justified in individual cases of the disease, such as in hospitalized and highly reproductive valued animals.

## Conclusions

We conclude that all three treatments administered to sheep affected by sub-clinical pregnancy toxaemia were able to restore normal blood glucose and ß-hydroxybutyrate concentrations. We consider that per os administration of 100 ml glycerol together with propylene glycol to each sheep every 12 h was the most interesting treatment, since it normalises the aforementioned biochemical parameters in a shorter period of time.

## Endnotes

Acetolena® Santa Elena, Uruguay

Dextrolena®, Santa Elena, Uruguay

Caninsulin®, Intervet, Argentina

Ranbut®, Randox Laboratories, Crumlin, Antrim, UK

Glucose Liquicolor®, Human Diagnostics, Wiesbaden, Germany

Humalyzer Junior photometer, Human Diagnostics, Wiesbaden; Germany

Statistica 6.0 (Stat Soft Inc, Tulsa, OK, USA, 2001).

## Abbreviations

OPT: Ovine pregnancy toxaemia
BOHB: ß-hydroxybutyrate
G: Glycerol
PG: Propylene glycol
